# Supplementary material for: Contribution of Live Video to Physicians’ Remote Assessment of Suspected COVID-19 Patients in an Emergency Medical Communication Centre: A Retrospective Study and Web-Based Survey
Source: Int J Environ Res Public Health. 2023 Feb 14;20(4):3307. doi: 10.3390/ijerph20043307 (PMC9959421; doi:10.3390/ijerph20043307)
Supplement: Supplementary file 1 [file ijerph-20-03307-s001.zip › Table S1.pdf]

Table S1: Web-based survey questionnaire: The value of live video calling in the context of assessments of suspected or confirmed COVID19 patients.

	Questions et réponses (FR)	Questions and answers (EN)
1	**Numéro d'évènement**	**Event Number**
2	**Date et heure de l'appel vidéo en direct**	**Date and time of the live video call**
3	**Nom du médecin***	**Name of physician**
4	**Sexe du patient (Homme /Femme)**	**Gender of patient (Male/Female)**
5	**Age du patient**	**Age of patient**
6	**Quel est le contexte de l'appel vidéo en direct ?** (une seule réponse)<br><br>- Patient seul à domicile<br>- Patient à domicile, avec présence d'une autre personne sur place<br>- Patient à domicile, avec un professionnel de santé sur place (hors présence d'une ambulance)<br>- Patient déjà dans un centre médical (hors présence d'une ambulance)<br>- Ambulance déjà sur place<br>- Ambulance et SMUR déjà sur place	**What is the context of the live video call?** (only one possible answer)<br><br>- Patient alone at home<br>- Patient at home, with another person present<br>- Patient at home, with a health professional on site (not including an ambulance)<br>- Patient already in a medical centre (without an ambulance)<br>- Ambulance already on site<br>- Ambulance and SMUR already on site
7	**Quelle est votre rôle* ?** (une seule réponse)<br><br>- Médecin trieur 2ème ligne<br>- Médecin effectuant un rappel à 24h<br>- Régulateur d'urgence<br>- Etudiants en médecine de 1ère ligne<br>- Etudiants effectuant un rappel à 24h<br>- Autre	**What is your role*?** (only one possible answer)<br><br>- Physician 2nd line<br>- Physician carrying out a 24-hour recall<br>- Emergency Medical Dispatcher<br>- 1st line medical students<br>- Student 24-hour recall<br>- Other
8	**Pour quelle raison avez-vous utilisé la vidéo en direct ?** (une seule réponse)<br><br>- Difficulté à communiquer<br>- Evaluation du contexte psycho-social<br>- Evaluation de l'état général du patient<br>- Evaluation de la respiration du patient<br>- Evaluation des paramètres vitaux (hors respiration)<br>- Evaluation de l'électrocardiogramme ou d'un autre examen paraclinique<br>- Autre	**Why did you use live video?** (only one possible answer)<br><br>- Difficulty to communicate<br>- Assessment of psycho-social context<br>- Assessment of patient's general condition<br>- Assessment of the patient's breathing<br>- Assessment of vital parameters (excluding respiration)<br>- Assessment of electrocardiogram or other paraclinical examination<br>- Other

9	**Selon vous, quelle est l'acceptation de l'utilisation par la personne en ligne ?** (une seule réponse)<br><br>- Pauvre<br>- Médiocre<br>- Acceptable<br>- Bon<br>- Très bon<br>- Excellent<br>- Autre	**What do you think is the acceptance of the use by the person online?** (only one possible answer)<br><br>- Poor<br>- Poor<br>- Acceptable<br>- Good<br>- Very good<br>- Excellent<br>- Other
10	**Comment jugez-vous la facilité d'utilisation de l'outil vidéo en direct (de votre côté) ?** (une seule réponse)<br><br>- Pauvre<br>- Médiocre<br>- Acceptable<br>- Bon<br>- Très bon<br>- Excellent<br>- Autre	**How do you rate the ease of use of the live video tool (on your side)?** (only one possible answer)<br><br>- Poor<br>- Poor<br>- Acceptable<br>- Good<br>- Very good<br>- Excellent<br>- Other
11	**Quel problème technique avez-vous rencontré ?** (une seule réponse)<br><br>- Echec réception SMS<br>- Impossibilité d'activer la caméra<br>- Ligne de mauvaise qualité<br>- Ligne interrompue<br>- Absence d'image<br>- Test<br>- Autre	**What technical problem did you encounter?** (only one possible answer)<br><br>- Failed to receive SMS<br>- Impossible to activate the camera<br>- Poor quality line<br>- Line interrupted<br>- No image<br>- Test**<br>- Other
12	**Quel problème d'utilisation avez-vous rencontré ?** (une seule réponse)<br><br>- Coupure du téléphone (audio)<br>- Coupure de la vidéo<br>- Autre	**What user problem did you encounter?** (only one possible answer)<br><br>- Telephone (audio) cut off<br>- Video cut off<br>- Other
13	**Quelle décision avez-vous prise à la suite de votre évaluation avec la vidéo en direct ?** (une seule réponse)<br><br>- Envoi d'une ambulance<br>- Envoi d'un médecin de garde à domicile<br>- Patient laissé sur site avec conseils<br>- Conseil de consultation par ses propres moyens	**What decision did you make as a result of your assessment with live video?** (only one possible answer)<br><br>- Dispatching an ambulance<br>- Dispatching an on-call doctor to the home<br>- Patient left on site with advice<br>- Advised to consult by own mean
14	**Quelle a été votre décision lorsque l'ambulance est déjà sur site ?** (une seule réponse)	**What was your decision when the ambulance is already on site?** (only one possible answer)

	- Transport en ambulance<br>- Patient laissé sur site<br>- Patient laissé sur site et envoi ultérieur d'un médecin de garde<br>- Patient laissé sur site avec conseil de consultation par ses propres moyens	- Transport by ambulance<br>- Patient left on site<br>- Patient left on site with subsequent dispatch of a physician on call<br>- Patient left on site with advice to consult by own means
**15**	**Est-ce-que la vidéo en direct a influencé votre décision ?** (une seule réponse)<br><br>- Oui<br>- Non<br>- Vidéo impossible ou de qualité insuffisante	**Did the live video influence your decision?** (only one possible answer)<br><br>- Yes<br>- No<br>- Video not possible or of insufficient quality
**16**	**Selon vous, quel a été l'apport de la vidéo dans votre décision ?** (question ouverte)	**In your opinion, how did the video influence your decision?** (open question)
