# Supplementary figures and images for: Contribution of Live Video to Physicians’ Remote Assessment of Suspected COVID-19 Patients in an Emergency Medical Communication Centre: A Retrospective Study and Web-Based Survey
Source: Int J Environ Res Public Health. 2023 Feb 14;20(4):3307. doi: 10.3390/ijerph20043307 (PMC9959421; doi:10.3390/ijerph20043307)

**Figure S1:** Monthly flow of remote assessments performed by physicians.

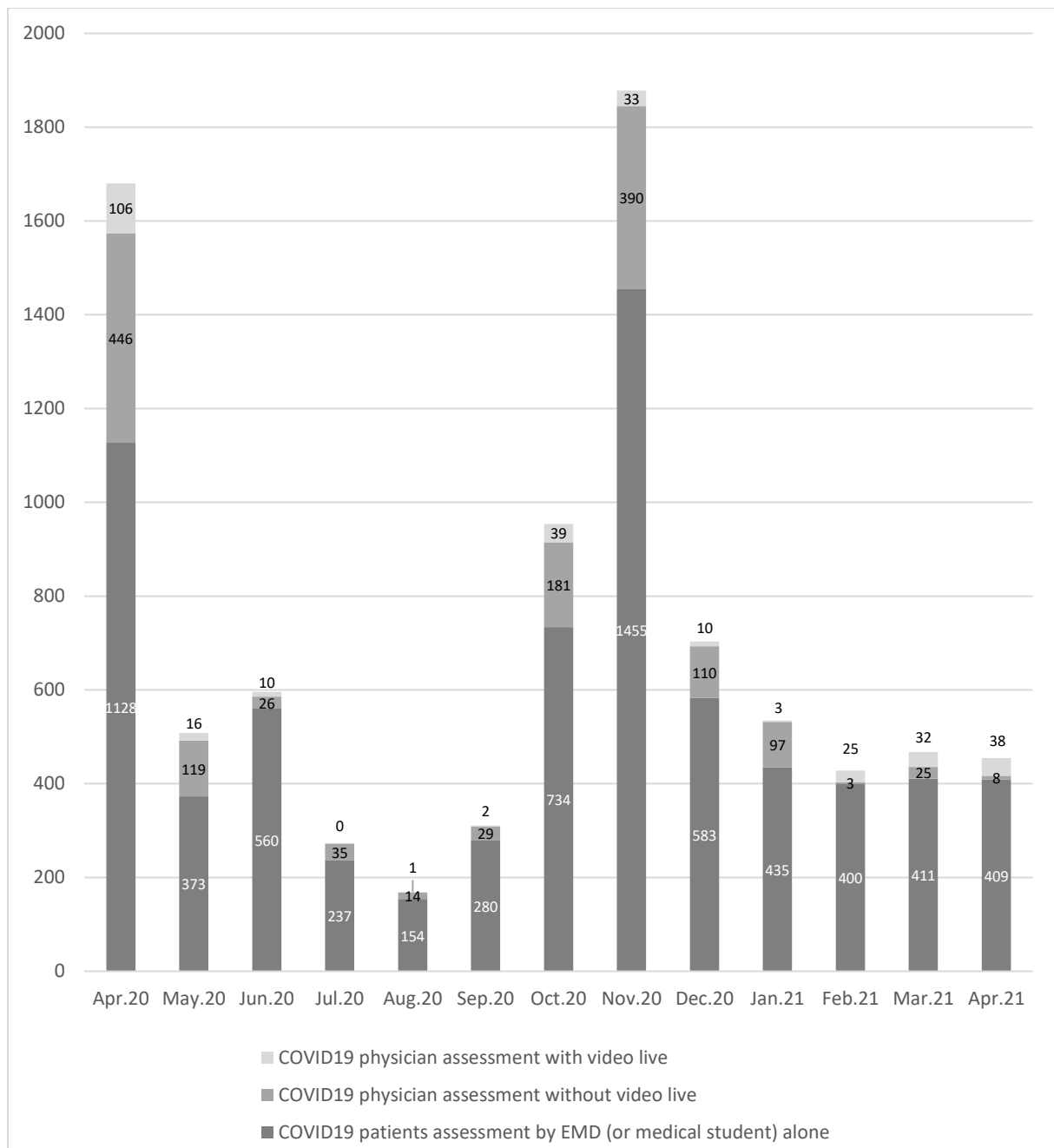

Supplement: Supplementary file 1 [file ijerph-20-03307-s001.zip › Figure S1.pdf]
